# Development and Validation of a Self-Administered Semiquantitative Food Frequency Questionnaire Focused on Gut Microbiota: The Stance4Health-FFQ

**DOI:** 10.3390/nu16234064

**Published:** 2024-11-26

**Authors:** Annarita Formisano, Marika Dello Russo, Paola Russo, Alfonso Siani, Daniel Hinojosa-Nogueira, Beatriz Navajas-Porras, Ángela Toledano-Marín, Silvia Pastoriza, Telmo Blasco, Alberto Lerma-Aguilera, María Pilar Francino, Francisco Javier Planes, Verónica González-Vigil, José Ángel Rufián-Henares, Fabio Lauria

**Affiliations:** 1 Institute of Food Science, National Research Council of Italy, 83100 Avellino, Italy; annarita.formisano@isa.cnr.it (A.F.); marika.dellorusso@isa.cnr.it (M.D.R.); paola.russo@isa.cnr.it (P.R.); alfonso.siani@isa.cnr.it (A.S.); 2 Department of Precision Medicine, University of Campania “L. Vanvitelli”, 80138 Naples, Italy; 3 Department of Nutrition and Food Science, Institute of Nutrition and Food Technology, Biomedical Research Centre, University of Granada, 18071 Granada, Spain; beatriznavajas@ugr.es (B.N.-P.); antolemarin@correo.ugr.es (Á.T.-M.); spdelacueva@ugr.es (S.P.); jarufian@ugr.es (J.Á.R.-H.); 4 Biomedical Engineering and Sciences Department, Tecnun School of Engineering, University of Navarra, 20018 San Sebastian, Spain; tblasco@unav.es (T.B.); fplanes@unav.es (F.J.P.); 5 Department of Genomics and Health, Fundació per al Foment de la Investigació Sanitària i Biomèdica de la Comunitat Valenciana (FISABIO-Salut Pública), 46020 València, Spain; alberto.lerma.aguilera@gmail.com (A.L.-A.); pilar.francino@fisabio.es (M.P.F.); 6 CIBER en Epidemiología y Salud Pública, 28029 Madrid, Spain; 7 Biomedical Engineering Center, University of Navarra, 31009 Pamplona, Spain; 8 Instituto de Ciencia de los Datos e Inteligencia Artificial (DATAI), University of Navarra, 31009 Pamplona, Spain; 9 Gestión de Salud y Nutrición S.L., 33003 Oviedo, Spain; vgonzalez@gsnsoft.es; 10 Instituto de Investigación Biosanitaria ibs. GRANADA, Universidad de Granada, 18012 Granada, Spain

**Keywords:** food frequency questionnaire, diet, validity, gut microbiota, Spain

## Abstract

**Background/Objectives**: Diet significantly influences gut microbiota (GM), with variations in GM responses linked to the type and quantity of food consumed. These variations underscore the need for personalized nutrition. The Stance4Health (S4H) project developed the S4H Food Frequency Questionnaire (S4H-FFQ) and the i-Diet S4H app to assess dietary intake of foods affecting GM. This study aimed to validate the S4H-FFQ against the validated I.Family-FFQ and the i-Diet S4H app; **Methods:** The S4H-FFQ, with 200 food items across 14 food groups, evaluates dietary intake over the past month. Qualitative validation compared food group consumption frequencies from the S4H-FFQ and the I.Family-FFQ, while quantitative validation assessed nutrient and energy intake using the i-Diet S4H app. The S4H-GM score, a measure of GM-relevant food consumption, was evaluated through the S4H-FFQ and i-Diet S4H app; **Results**: Pearson correlations between the S4H-FFQ and the I.Family-FFQ ranged from 0.3 to 0.7 and were statistically significant across all the food groups. Quantitative validation showed lower but consistent correlations, comparable with other studies, confirming the S4H-FFQ’s ability to estimate food intake. A positive correlation was also found between the S4H-GM scores from the S4H-FFQ and the i-Diet S4H app (*p* < 0.001); **Conclusions**: The S4H-FFQ is a reliable tool for assessing dietary patterns that influence GM. Its application in nutritional studies can enhance personalized nutrition and support future research aimed at optimizing GM and improving health outcomes.

## 1. Introduction

Emerging evidence suggests that gut microbiota and their products play a pivotal role in human health, especially in relation to immune-related diseases and metabolic disorders like Type 2 diabetes, obesity, and cardiovascular disease [1]. The composition and diversity of the microbiota are shaped by various factors including life stages, genetic characteristics of the host, and environmental factors. Among the latter, diet plays a significant role in modulating the gut microbiota [2]. The microorganisms in the GM exhibit varied responses intricately linked to the type and quantity of food consumed [3]. In addition, the microbiota has been shown to play a key role in the inter-individual response of individuals to the same dietary pattern [4]. This heterogeneous response of individuals to the same dietary pattern increases the need for a personalized approach to nutrition [5]. Although personalized nutrition is a relatively young discipline, it is fundamental to take into account the relationship of the individual with his diet and the different factors that surround him, such as his genetic characteristics or the microbiota [6]. Consequently, a precise and accurate individual dietary assessment is a fundamental aspect of nutritional intervention studies aimed at identifying the complex relationship between diet, gut microbiota, and health status [7].

Although several dietary assessment methods are available, the selection of the most appropriate tool depends on the objectives of the study, the resources available, the population under study and the specific study design [8]. Food Frequency Questionnaires (FFQs) are commonly used as a primary dietary assessment method in epidemiological studies due to their low cost, ease of use for participants, and ability to capture long-term dietary patterns [9]. By assessing food intake over months or years, rather than just focusing on short-term consumption, the FFQ offers a more comprehensive understanding of dietary influence on health outcomes [10]. Despite limitations in estimating absolute dietary intakes, when properly validated against dietary records or biomarkers, they can reliably reflect the habitual diet of a population [11,12].

The primary focus of the Stance4Health (Smart Technologies for personAlised Nutrition and Consumer Engagement) project was to develop and provide standardized instruments for assessing dietary habits and intake among European citizens. The objective was to contribute nutritional guidance that could positively influence gut microbiota composition and functionality. As part of the project, a nutritional intervention was developed to validate specific personalized nutrition strategies aimed at enhancing human health through the modulation of the gut microbiota [13]. For this purpose, a new set of dietary assessment instruments was designed and developed, comprising two complementary methods. The i-Diet S4H app (v.02.24) [14] was developed to foster balanced nutrition and healthy habits by providing personalized recommendations, considering individual characteristics, physical activity factors, dietary preferences, and gut microbiota nutritional requirements. The app features a “Gut Microbiota score” (S4H-GM score) within its menu-generation system. This score, based on different algorithms developed within the framework of the S4H project [15,16], ranks foods according to their ability to nourish the gut microbiota healthily. Studies suggest that fermented foods, plant-based diets, high-fiber intake, and polyphenol-rich foods significantly influence gut microbiota [17]. Diets centered around these foods may foster a diverse and beneficial microbial environment, essential for overall health and disease prevention [18,19]. Therefore, by integrating the S4H-GM score into the app’s menu-generation system, it can provide recommendations that not only meet nutritional requirements but also promote healthy nourishment of the gut microbiota.

On the other hand, validated and reproducible FFQs are available and well-documented for various populations, including general adults, the elderly, and children [20,21,22]. However, none of them aligned with the specific objectives of the S4H project. Thus, in the framework of the Stance4Health project, a semiquantitative food frequency questionnaire (S4H-FFQ) was developed to record the consumption frequency of 200 pan-European food items that could potentially affect gut microbiota composition and functionality. Therefore, the present study aimed to evaluate the ability of the S4H-FFQ to estimate proxy-reported intakes of gut microbiota-related foods in an adult Spanish population and compare food estimates with those obtained from the validated and reproducible I.Family-FFQ and the i-Diet S4H app. The S4H-FFQ, integrated with the Food Composition Tables (FCTs) developed for the S4H project, offers a reliable and adaptable tool for assessing dietary patterns across populations. By enabling robust comparative nutritional research, it supports the development of targeted interventions and policies that can influence health outcomes in Europe.

## 2. Materials and Methods

### 2.1. Study Population

The validation study sample was recruited in Oviedo by the University of Granada, under the framework of the S4H project [13]. The overall objective of S4H was to develop a complete Smart Personalized Nutrition (SPN) service based on the use of mobile technologies as well as tailored food production able to optimize GM activity and long-term consumer engagement. The study was conducted in accordance with the Declaration of Helsinki and approved by the Ethics Committee for Investigation of the University of Granada (Protocol number 1080/CEIH/2020, date 10 June 2020). Informed written consent was obtained from each participant. The project was registered with the ISRCTN registry (https://www.isrctn.com/ISRCTN63745549, accessed on 11 November 2024), registration number ISRCTN63745549.

A total of 133 apparently healthy adult individuals (both sexes), aged 20–65 years, with a body mass index (BMI) between 20 and 28 kg/m^2^, and at a stable weight, were included in the present study. In Table 1, the study population is presented. Participants with a diagnosis of chronic gastrointestinal disorders, celiac disease, or chronic diseases, present pregnancy or lactation, recent inflammation, medically prescribed diet, antibiotic treatment, intake of antioxidants, pre- or probiotic supplements, intense physical activity, and high alcohol consumption were excluded [13].

At the first meeting between the S4H staff and people interested in participating in the study, information about participants’ age, sex, anthropometry, dietary habits, health status, and contact details were collected to select people with the identified inclusion/exclusion criteria. Eligible participants were asked to complete the informed consent form plus online questionnaires on their lifestyle, bowel movements, and dietary habits (S4H-FFQ and I.Family-FFQ) and provide a fecal sample collection. Fecal samples were collected from all participants at each study period. These samples were used to obtain gut microbiota composition data through 16S rRNA gene sequencing. The i-Diet S4H app was used to record food intake. Details about the study protocol can be found elsewhere [13].

### 2.2. Study Design

Three clinical evaluations of the study populations were performed: at the beginning (T0), after 6 weeks (T1), and after 12 weeks at the end of the nutritional interventions (T2). At T0 and T2, dietary information through two FFQs, sent to the participants by e-mail in Google Forms format, was collected. The two FFQs used in the study were: the I.Family-FFQ [23] and the S4H-FFQ, which was developed for use in the Stance4Health study with a particular emphasis on understudied food groups (such as spices) and their potential utility as a tool to support the modulation of the gut microbiota.

The validity of the S4H-FFQ was assessed by: (i) Comparing different food groups’ frequency consumption derived from the two FFQs (qualitative validation); (ii) Comparing the energy and nutrient intakes derived from the S4H-FFQ against the i-Diet S4H app (quantitative validation); (iii) Comparing the S4H-GM score calculated by the S4H-FFQ and the i-Diet Stance4Health app.

**Figure 1:**
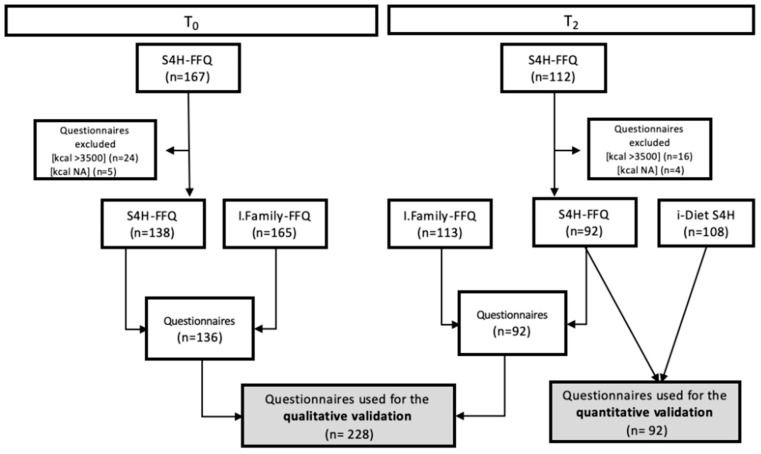
Flow chart of questionnaires included in final analysis.

The flow chart of the selection process is shown in Figure 1. FFQs reporting total energy intakes below 500 kcal or above 3500 kcal were deemed implausible and excluded from the analysis. For the qualitative validation study, questionnaires filled out at T0 and T2 were included. For the quantitative validation study, only the questionnaires filled out at T2 were included, as, during this wave, we collected nutritional information using both the S4H-FFQ and i-Diet S4H app for the same period.

### 2.3. Nutritional Assessment Tools

The semi-quantitative online S4H-FFQ was designed as a screening tool to assess eating behaviors associated with GM composition in the S4H project. The S4H-FFQ was based on the FFQ developed and validated in the EPIC study [21,22,24], and on the FFQ of the PREDIMED study [25] developed and validated by Martin-Moreno et al. [26]. The two questionnaires have been expanded to include various foods that can modulate the gut microbiota, as well as alternative foods like seaweed, foods commonly consumed in Spain, such as fermented vegetables, and spices, which are not always included in this type of questionnaire. It consists of 200 food items separated into 14 food groups: “vegetables”, “tubers”, “fruits”, “nuts and spices”, “legumes”, “cereals”, “milk and milk products”, “oils and fats”, “eggs, meats and meat derivatives”, “fish and fish products, seafood and crustaceans”, “pastry”, “beverages without alcohol”, “alcoholic beverages”, “miscellaneous”. The frequencies of consumption were reported on an incremental scale with nine levels (Never or hardly never, 1–3 times per month, 1 time a week, 2–4 times a week, 5–6 times a week, 1 time per day, 2–3 times per day, 4–6 times per day, 6 or more times per day). Each item reported standard portion size in grams or milliliters and in common household portions (plate, cup, tablespoon, teaspoon, glass, piece, slice, pinch).

For the qualitative validation of the S4H-FFQ, the self-administered I.Family-FFQ was used. It is a qualitative questionnaire designed to assess eating behaviors, during a typical week over the previous month, associated with the risk of being overweight, obesity, and general health. The FFQ was found to provide reproducible and valid data [20,23,27]. It includes 43 Pan-European food items clustered into 15 food groups according to their nutritional profiles: “vegetables”, “fresh fruits”, “drinks”, “breakfast cereals”, “milk”, “yoghurt”, “fish”, “meat and meat products”, “eggs and mayonnaise”, “meat replacement products and soy products”, “cheese”, “spreadable products”, “plant oil for cooking and/or salads”, “cereal products”, “snacks”. Participants indicated their frequency of consumption over the previous month. The frequencies of consumption were reported on an incremental scale with seven levels (Never/less than once a week, 1–3 times a week, 4–6 times a week, 1 time per day, 2 times per day, 3 times per day, 4 or more times per day).

The FFQ was adapted and validated for use with a pediatric population [28]. Furthermore, the questionnaire is provided as Supplementary Material (Excel Sheet S1) and is available in multiple languages, including Spanish, English, Greek, German, and Italian.

For the quantitative validation, the i-Diet S4H app [14] was used. This instrument was able to record food intake throughout the nutritional intervention. Participants recorded the quantity and type of foods and beverages consumed during the day, utilizing standardized photographs to ensure consistency in portion estimation. The app’s integrated Food Composition Tables (FCTs) [29] were linked to each food item or recipe, facilitating the calculation of the corresponding nutritional values.

The S4H-GM score defines the impact of food consumption on the metabolic function of an individual’s gut microbiota, which can produce beneficial and harmful metabolites for the host’s physiology. This score can be positive or negative, reflecting the healthy or unhealthy effect of ingested micronutrients on the metabolic function of the gut microbiota. Specifically, the S4H-GM score was calculated based on the S4H-GM score per ingredient, which determines the impact of 100 g of an ingredient on the metabolic function of the gut microbiota. By considering the quantities of different ingredients consumed per individual and day, the S4H-GM score was derived as a weighted sum of the S4H-GM score per ingredient. To assess the impact of 100 g of a specific ingredient on the metabolic function of an individual’s gut microbiota, a genome-scale metabolic model was built based on: (i) AGREDA [15], high-quality metabolic reconstruction of the human gut microbiota; (ii) 16S rRNA gene sequencing data from individual’s fecal samples; and (iii) input micronutrient composition of 100 g of a specific ingredient. Then, an optimization algorithm was applied to predict the net production of key metabolites for human health [30]. Finally, the S4H-GM score was obtained for the ingredient by normalizing and averaging the production of these metabolites, considering that beneficial and harmful metabolites take a positive and negative sign in the score, respectively. This score was integrated into the app’s recommendation engine, prioritizing the most beneficial ingredients while minimizing the use of less favorable ones. The recommendation engine focused on recommending those combinations that not only meet the user’s nutritional needs and preferences but also promote a microbiota-friendly diet. The same methodology was followed, but using the FFQ-S4H data instead, for those from the i-Diet S4H app.

### 2.4. Statistical Analyses

Descriptive and association analyses were performed using IBM SPSS Statistics (Version 29.0, IBM Corp., Armonk, NY, USA), the R programming language v. 4.0.1 (R Foundation for Statistical Computing, Vienna, Austria) and RStudio software environment v. 2024.04.2+764 (Posit Software, PBC, Boston, MA, USA). Before statistical tests were carried out, the normality of the data in this study was tested using the Anderson–Darling test with a 0.05 significance level. Considering that correlations in dietary validation studies typically range from 0.3 to 0.7, the population under study has sufficient power (>99%) to detect a correlation of r = 0.5 with α = 0.05. Even for smaller correlations (e.g., r = 0.3), the sample size is likely adequate to achieve at least 80% power. Descriptive analysis was used to obtain the frequency, percentage, mean, and standard deviation of anthropometric and dietary intake data.

The frequency of consumption of the 15 food groups of the I.Family-FFQ was compared to the frequency of consumption of the same groups of the S4H-FFQ. To do this, the different items were grouped into food groups following the structure of the I.Family-FFQ. Except for “Breakfast”, “Milk”, and “Spread”, the food groups created from the S4H-FFQ contained a large number of items compared to those created for the I.Family-FFQ. For each food item, the daily consumed amount was calculated by multiplying the consumption frequency by the portion size in grams. Energy and nutrient intakes were estimated by multiplying the daily consumed amount by the energy or nutrient content per 100 g of the food item. Finally, total daily energy and nutrient intakes were standardized per 1000 kcal.

The FCTs specifically developed in the framework of the S4H project [29] were used to derive energy and nutrient intakes from the dietary data of the collected food items. A total number of 228 questionnaires was considered for this analysis, including questionnaires collected at T0 and T2 (see the flow chart in Figure 1). FFQs with total energy intake <500 and >3500 kcal from the S4H-FFQ were considered implausible and excluded from the analysis. No individuals with a total energy intake <500 kcal were found.

Energy and macronutrient intake derived from the S4H-FFQ were compared with the i-Diet S4H app using Pearson’s correlation coefficient. An average intake from the last 4 weeks of use of the nutritional i-Diet S4H app was compared with the intake calculated from the S4H-FFQ (which covered the same period). A total of 92 questionnaires were considered for this analysis, including questionnaires collected at T2 (see the flow chart in Figure 1).

Pearson’s correlation was also used to correlate the S4H-GM score calculated based on the S4H-FFQ and the i-Diet Stance4Health for the individual items and items grouped into corresponding food groups. In addition, a Bland–Altman plot was used to graphically examine the agreement between the two FFQs for the frequency of consumption of the considered groups, and between the S4H-FFQ and the i-Diet S4H app for energy and macronutrient intake [31]. Variables were log-transformed to obtain a normal distribution. The Bland–Altman plots showed the differences in intake between the measurement methods by the two instruments (y-axis), against the mean intake of the two measures (x-axis). Cross-classification analysis was done by classification of participants into quintile categories based on the frequencies and dietary intake data from both the FFQs and the S4H-FFQ and the i-Diet S4H app.

## 3. Results

In Table 1 the population characteristics of 133 Spanish men and women involved in the present analysis are shown. Participants had a mean ± SD age of 44.4 ± 10.1 years, and 65% were female. Participants completed the I.Family-FFQ, S4H-FFQ, and used the i-Diet S4H app during the nutritional intervention of the S4H study.

### 3.1. Comparison of Different Food Groups’ Frequency Consumption Derived from the Two FFQs

**Figure 2:**
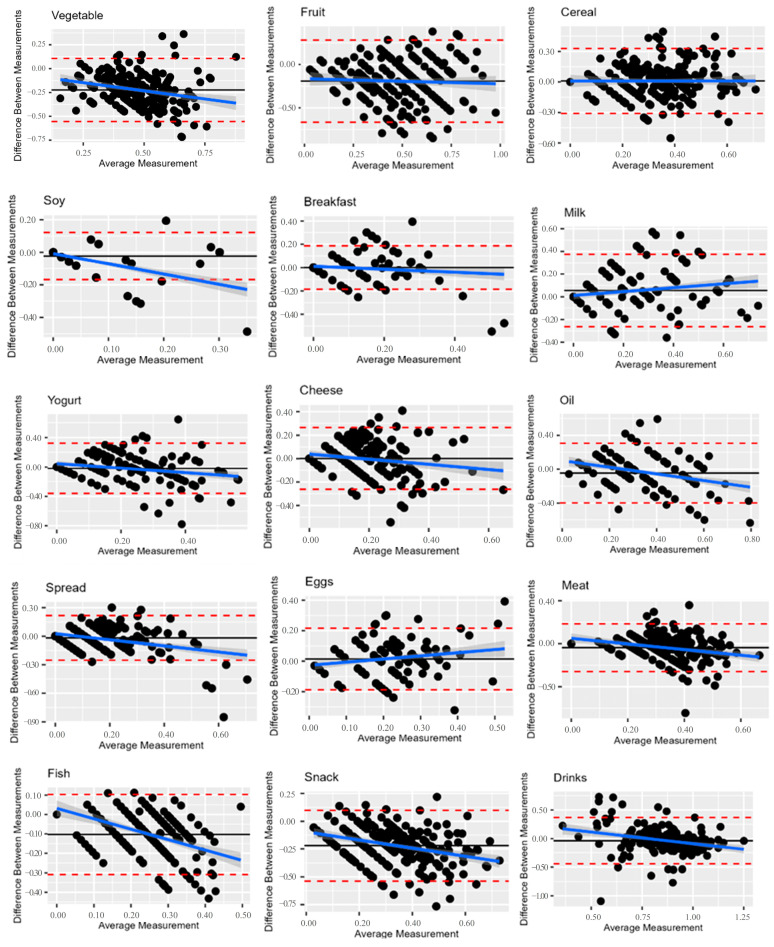
Bland–Altman plots between the S4H-FFQ and I.Family-FFQ for different food groups’ frequency consumption. The blue line represents the regression line of proportional bias, while the black line represents the mean (constant bias), and the dashed lines indicate 95% confidence intervals.

Based on Bland–Altman graphs in Figure 2, a decreasing trend was observed in most of the graphs, which could indicate a proportional error, i.e., positive differences for small values, and vice versa, negative differences for large values. Means were calculated as F_α IFamily-FFQ_ − F_α S4H-FFQ_, so the S4H-FFQ tended to overestimate the frequency of consumption as intake frequency increased. This was true with the exclusion of the “Milk” and “Eggs” food groups and was probably due to the different number of items between the two FFQs. The total intake of the food groups generally increased proportionally to the number of items, and the S4H-FFQ is more detailed than the I.Family-FFQ. A different scenario occurred for “Drinks”, for which the point cloud was more concentrated.

The I.Family-FFQ and S4H-FFQ showed a relatively comparable mean of the frequency of consumption of the 15 food groups (Table 2). Pearson correlations ranged from 0.3 to 0.7 and were statistically significant for all the food groups. High values were observed for the food groups for which the number of items was similar (Breakfast, Milk, Spread). The lowest correlation was observed for “Drinks”. In addition, the S4H-FFQ classified ≥ 67% of the participants in the same or adjacent quintile as the I.Family-FFQ for all food groups under study (Table 3). Misclassification ≥6% in the extreme quintile did not occur for any of the nutrients under study.

### 3.2. Comparison of the Food Frequency Consumption Derived from the S4H-FFQ Against the i-Diet S4H App

**Figure 3:**
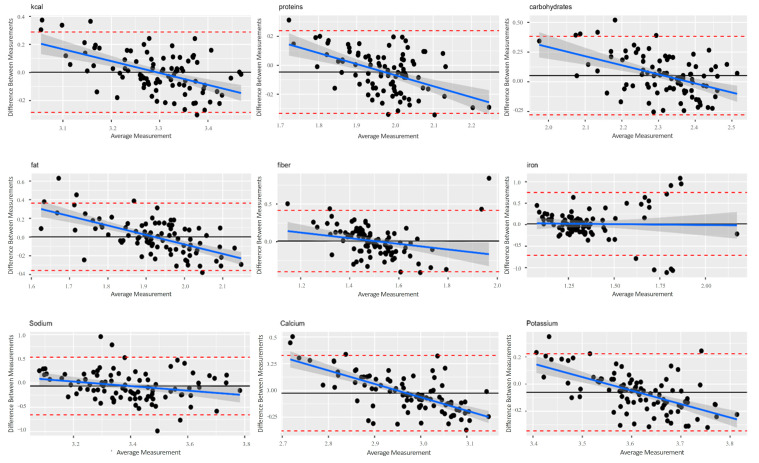
Bland–Altman plots between the S4H-FFQ and i-Diet S4H app for energy and nutrients. The blue line represents the regression line of proportional bias, while the black line represents the mean (constant bias), and the dashed lines indicate 95% confidence intervals.

In Figure 3, the Bland–Altman graphs for the different nutrients show a decreasing trend with the exclusion of the “iron” graph. A possible overestimation of S4H-FFQ was shown at higher intakes. The S4H-FFQ and i-Diet S4H app showed poor correlation values. Pearson correlations ranged from 0.2 to 0.3 for all macronutrients (g/d) and from 0.1 to 0.2 for the considered micronutrients (Table 4). Moreover, the S4H-FFQ classified ≥ 50% of the participants in the same or adjacent quintile as the i-Diet S4H app for all nutrients under study (Table 5). Misclassification ≥8% in the extreme quintile did not occur for any of the nutrients under study.

### 3.3. Comparison of the S4H-GM Score Calculated Based on the S4H-FFQ and the i-Diet S4H App

**Figure 4:**
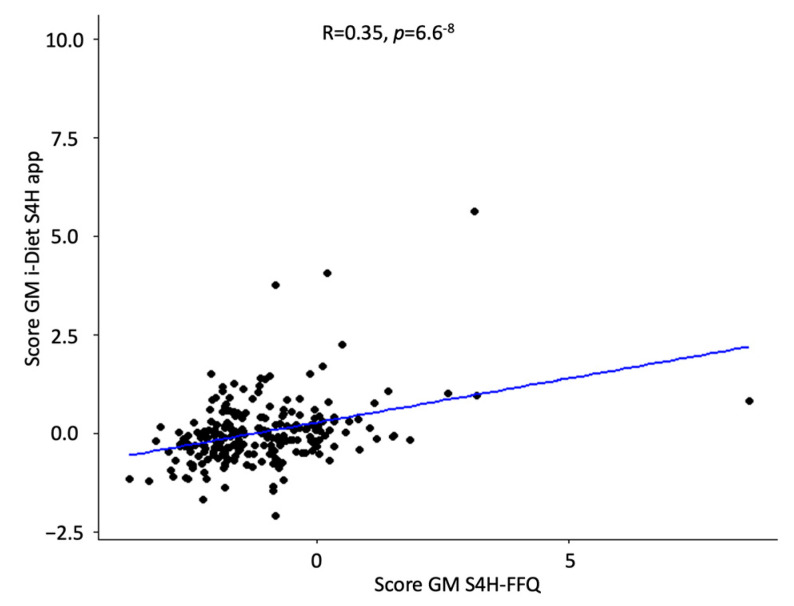
Correlation between the S4H-GM score calculated by the S4H-FFQ and the i-Diet S4H app.

Figure 4 shows a positive and significant correlation (R = 0.35, *p* < 0.001) between the S4H-GM score calculated by the S4H-FFQ and the i-Diet S4H app.

**Figure 5:**
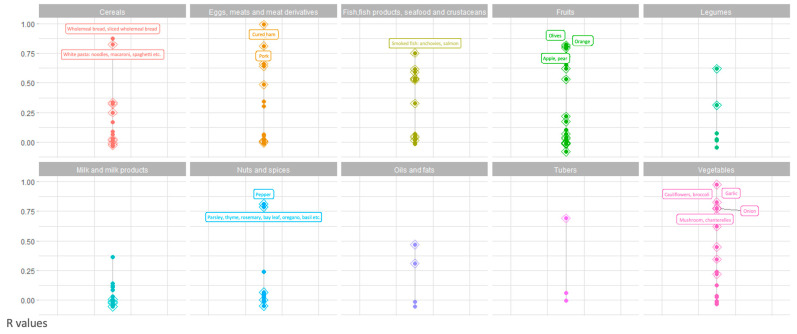
Correlation between the S4H-GM scores of individual items from the i-Diet S4H app and those from the S4H-FFQ grouped into corresponding food groups. Diamonds indicate *p*-value < 0.05.

Figure 5 shows significant positive correlations between the S4H-GM scores of individual items from the i-Diet S4H app and those from the S4H-FFQ grouped into corresponding food groups. Data points with correlations exceeding 0.75 are indicated in the figure, with statistically significant correlations (*p* < 0.05) represented by diamonds. The “Pastry,” “Alcoholic beverage”, “Non-alcoholic beverage” and “Miscellaneous” groups were removed because all correlations were below 0.25.

## 4. Discussion

This study describes the validation of the online semi-quantitative S4H-FFQ. It contains 200 food items grouped into 14 food groups and was designed to report on the dietary intake of an adult population in the last month. The questionnaire was developed to investigate the frequency of consumption of foods capable of influencing gut microbiota composition and functionality of the participants involved in the nutritional intervention of the S4H project. In this sense, research indicates that diets rich in animal proteins tend to elevate levels of bile-tolerant microorganisms while reducing the abundance of saccharolytic microorganisms [32,33]. In contrast, plant protein-based diets increase the levels of lactobacilli and bifidobacteria [34]. Dietary fiber from fruits, vegetables, and whole grains plays a vital role in fostering a robust gut microbial ecosystem by stimulating the production of short-chain fatty acids (SCFAs). This process bolsters immune defenses and maintains overall gut health [35,36]. A high intake of dietary fat, particularly saturated fatty acids, appears instead to promote the development of pro-inflammatory intestinal microbiota, resulting in increased intestinal permeability, elevated circulating levels of lipopolysaccharides, and the onset of chronic-degenerative diseases [37]. In addition, the gut microbiota has been shown to play a key role in the inter-individual response of individuals to the same dietary pattern [4]. This heterogeneous response increases the need to develop what is known as personalized or precision nutrition [5,38]. In this context, the i-Diet S4H app algorithm, utilizing the S4H-GM score, was designed to recommend foods with the greatest positive impact on gut microbiota. However, the menu creation process did not solely focus on maximizing these scores; it also prioritized ensuring that the user’s nutritional needs were fully met and that the recommendations aligned with the user’s preferences to support long-term adherence. The primary goal was to design menus that satisfy dietary requirements, incorporate appropriate food matrices, and achieve a positive impact on the microbiota, all while respecting the user’s preferences and lifestyle.

Developing and validating dietary assessment methods, such as the S4H-FFQ, is crucial to accurately measure food intake affecting gut microbiota composition in nutritional studies. From a nutritional point of view, the S4H-FFQ was validated against two validated instruments: the I.Family-FFQ and the i-Diet S4H nutrition app. As shown in Table 2, correlation analysis revealed levels of agreement from acceptable to very good between the S4H-FFQ and the qualitative I.Family-FFQ. However, in line with similar nutritional validation studies, the quantitative analysis showed low correlation values (Table 4). Specifically, only total energy intake and kcal from fat showed acceptable correlation values (r = 0.3) with significant *p*-values. Related to this, a recent meta-analysis showed that using mobile apps to record dietary data could underestimate energy and macronutrient intakes with regard to more traditional methods [39]. These aspects, in addition to the hypothetical influence of social desirability bias, and psychosocial factors on the accuracy of self-reported consumption of specific foods [40,41], could justify our results. Nevertheless, the newly developed S4H-FFQ was shown to have the potential to capture the usual diet of adult healthy individuals as reflected by its good correlation with the I.Family-FFQ. However, in line with the principal purpose of the S4H-FFQ, it should be emphasized that the evaluation of macro- and micro-nutrients was not the primary aim of the questionnaire, which was focused on the frequency of consumption of foods and beverages impacting gut microbiota composition at a population level.

In the era of personalized nutrition, the use of technology is essential for developing tailored dietary advice and tools [42]. In this sense, this study has placed value on the use and versatility of an FFQ. While it is true that the results for both correlations and quintile adequacy are low, we have to consider that, in this kind of study, achieving high correlations can be challenging due to inherent complexities in dietary intake measurement and reporting biases, also considering that this method relies on the memory of participants. Thus, low “r” values might be acceptable if they are supported by significant *p*-values, indicating a meaningful relationship despite being modest [11,43] (Table 3 and Table 4). Moreover, validation was performed with previously validated tools and in a large and well-structured personalized nutrition intervention [13,14,23].

The final assessment should always be made on the basis of information that takes into account dietary patterns that represent habitual combinations of foods and nutrients that cumulatively compose the diet. It is well-established that not only food composition but also cooking methods could play an important role in the modulation of the gut microbiota, due to chemical changes in foodstuffs during the cooking process [44,45,46]. The S4H-FFQ also includes a general question about the habitual methods used to prepare vegetables as well as potatoes, meat, and fish food groups. No specific questions were considered to record how consumed foods were cooked, preventing us from identifying this aspect on the different participants.

Finally, we used the S4H-GM score to confirm the validity of the FFQ. The statistically significant correlation between S4H-GM scores from the S4H-FFQ and the i-Diet S4H app, both considering the single foods items and items grouped into food groups (Figure 4 and Figure 5), reinforced the reliability of the dietary data collected through the S4H-FFQ. This confirmed the S4H-FFQ as an effective tool for capturing dietary patterns that may influence gut microbiota, making it valuable for gut microbiota-focused nutritional research.

A key strength of our study is the large sample size of participants, all of whom were apparently healthy and selected based on well-defined inclusion and exclusion criteria. Additionally, the newly developed S4H-FFQ, which includes a wide range of pan-European food items, can be easily adapted for use in other populations to examine dietary composition in nutritional studies. In addition, the S4H-FFQ has already been translated into several European languages and is associated with the S4H FCDB, one of the most comprehensive databases in terms of the number of harmonized foods, nutrients, and bioactive compounds included. It could be adapted and validated in various populations. Such a study could provide a reliable and standardized tool for comparative nutritional research, potentially influencing health outcomes at the European level. A limitation of the present study is that the results obtained on some food items, nutrients, and energy intake have been shown to be misreported in the S4H-FFQ compared to data from the i-Diet S4H app, and such results should be interpreted with caution. This limitation could be addressed by using metabolomic biomarkers, although only a few comprehensively validated biomarkers of food intake are available [47]. However, the use of the S4H-GM score reinforced the validity of the questionnaire.

## 5. Conclusions

The findings of the present study suggest that the S4H-FFQ is capable of providing acceptable food estimates for the population under study. Specifically, it demonstrates utility as an effective instrument in nutritional studies for assessing the consumption of specific foods and dietary habits, with potential implications for GM composition. This study emphasizes the potential of these tools in tackling emerging nutritional challenges, such as promoting the consumption of targeted foods to influence the GM. We view this tool as a breakthrough for future epidemiological studies on personalized nutrition aimed at optimizing GM and improving health outcomes. Furthermore, integrated with the S4H FCTs, the S4H-FFQ offers valuable insights for guiding evidence-based policies and improving public health interventions to address dietary challenges and enhance health outcomes across diverse European populations.

## Figures and Tables

**Table 1:** Characteristics of the population.

Characteristic	Values
Participants (n.)	136
Age (years)	44.4 (10.1)
Sex (female)	87 (65)
BMI (kg/m^2^)	24.8 (3.0)
n. S4H-FFQ (T0 + T2)	279
n. I.Family-FFQ (T0 + T2)	278
n. i-Diet S4H (T2)	108

Values are expressed as Mean (SD) or n (%). BMI: Body Mass Index.

**Table 2:** Pearson correlations of the frequency of consumption of the 15 food groups.

Food Group	RHO	*p*-Value
Vegetable	0.384	1.98 × 10^−9^
Fruit	0.425	2.02 × 10^−11^
Cereal	0.435	5.84 × 10^−12^
Soy	0.554	9.67 × 10^−20^
Breakfast	0.702	3.73 × 10^−35^
Milk	0.680	2.69 × 10^−32^
Yogurt	0.464	1.47 × 10^−13^
Cheese	0.459	2.97 × 10^−13^
Oil	0.482	1.11 × 10^−14^
Spread	0.714	8.82 × 10^−37^
Eggs	0.484	8.16 × 10^−15^
Meat	0.419	4.38 × 10^−11^
Fish	0.439	3.71 × 10^−12^
Snack	0.565	1.24 × 10^−20^
Drinks	0.303	3.28 × 10^−6^

**Table 3:** Comparison of quintiles distribution of the frequency of consumption of the 15 food groups estimated by the S4H-FFQ and the I.Family-FFQ.

Food Group	Same Quintiles (n.)	Adjacent Quintiles (n.)	Opposite Quintiles (n.)	Same Quintiles (%)	Adjacent Quintiles (%)	Opposite Quintiles (%)
Vegetable	66	95	4	29	42	2
Fruit	80	75	6	35	33	3
Cereal	78	75	13	34	33	6
Soy	57	171	0	25	75	0
Breakfast	179	40	0	79	18	0
Milk	87	96	0	38	42	0
Yogurt	63	117	2	28	51	1
Cheese	72	102	4	32	45	2
Oil	103	61	6	45	27	3
Spread	80	114	0	35	50	0
Eggs	91	72	6	40	32	3
Meat	82	90	6	36	39	3
Fish	71	86	2	31	38	1
Snack	78	92	3	34	40	1
Vegetable	87	90	11	38	39	5

**Table 4:** Pearson correlation of the energy and nutrients.

Nutrient	RHO	*p*-Value
Energy	0.2422	0.0200
Proteins	0.2601	0.0123
Carbohydrates	0.2293	0.0279
Fat	0.2404	0.0210
Fiber	0.2175	0.0373
Iron	0.1164	0.2690
Calcium	0.1194	0.2568
Potassium	0.2272	0.0294
Sodium	0.0672	0.5246

**Table 5:** Comparison of quintiles distribution of the energy and nutrients estimated by the S4H-FFQ and the and i-Diet S4H app.

Nutrient	Same Quintiles (n.)	Adjacent Quintiles (n.)	Opposite Quintiles (n.)	Same Quintiles (%)	Adjacent Quintiles (%)	Opposite Quintiles (%)
Energy	24	30	6	26	33	7
Proteins	22	24	2	24	26	2
Carbohydrates	18	35	5	20	38	5
Fat	23	34	4	25	37	4
Fiber	25	32	6	27	35	7
Iron	22	24	5	24	26	5
Calcium	27	25	4	29	27	4
Potassium	23	33	6	25	36	7
Sodium	21	36	7	23	39	8

## Data Availability

The data presented in this study are available on request from the corresponding author due to the privacy of participants.

## Supplementary Materials

The following supporting information can be downloaded at: https://www.mdpi.com/article/10.3390/nu16234064/s1, Excel S1: Food Frequency questionnaire Stance4Health.
